# Supplementary material for: Saccharomyces boulardii (CNCM I-745) improves intestinal damage in sepsis by remodeling intestinal flora structure
Source: Front Cell Infect Microbiol. 2025 Jul 31;15:1602792. doi: 10.3389/fcimb.2025.1602792 (PMC12350329; doi:10.3389/fcimb.2025.1602792)
Supplement: Supplementary file 1 [file DataSheet1.zip › summary/summary/8_Advanced_analyse/2_PICRUSt2_result/COG/Group.DW4_vs_BW4/PICRUSt2_COG_Group.DW4_vs_BW4_diff.pdf]

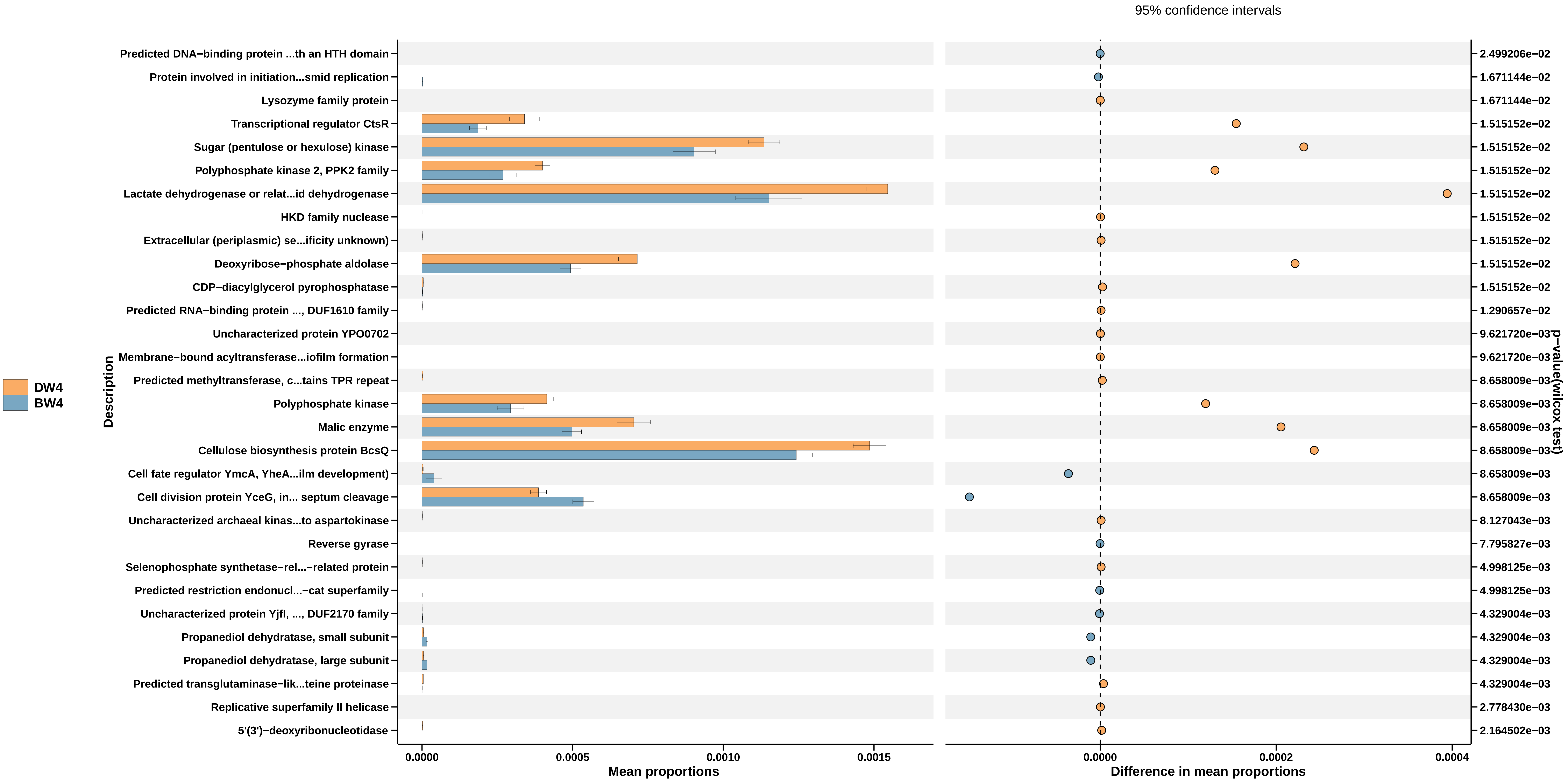
95% confidence intervals
DW4
BW4
Description
Predicted DNA−binding protein ...th an HTH domain
Protein involved in initiation...smid replication
Lysozyme family protein
Transcriptional regulator CtsR
Sugar (pentulose or hexulose) kinase
Polyphosphate kinase 2, PPK2 family
Lactate dehydrogenase or relat...id dehydrogenase
HKD family nuclease
Extracellular (periplasmic) se...ificity unknown)
Deoxyribose−phosphate aldolase
CDP−diacylglycerol pyrophosphatase
Predicted RNA−binding protein ..., DUF1610 family
Uncharacterized protein YPO0702
Membrane−bound acyltransferase...iofilm formation
Predicted methyltransferase, c...tains TPR repeat
Polyphosphate kinase
Malic enzyme
Cellulose biosynthesis protein BcsQ
Cell fate regulator YmcA, YheA...ilm development)
Cell division protein YceG, in... septum cleavage
Uncharacterized archaeal kinas...to aspartokinase
Reverse gyrase
Selenophosphate synthetase−rel...−related protein
Predicted restriction endonucl...−cat superfamily
Uncharacterized protein YjfI, ..., DUF2170 family
Propanediol dehydratase, small subunit
Propanediol dehydratase, large subunit
Predicted transglutaminase−lik...teine proteinase
Replicative superfamily II helicase
5'(3')−deoxyribonucleotidase
0.0000
0.0005
0.0010
0.0015
Mean proportions
0.0000
0.0002
0.0004
Difference in mean proportions
2.499206e−02
1.671144e−02
1.671144e−02
1.515152e−02
1.515152e−02
1.515152e−02
1.515152e−02
1.515152e−02
1.515152e−02
1.515152e−02
1.515152e−02
1.290657e−02
9.621720e−03
9.621720e−03
8.658009e−03
8.658009e−03
8.658009e−03
8.658009e−03
8.658009e−03
8.658009e−03
8.127043e−03
7.795827e−03
4.998125e−03
4.998125e−03
4.329004e−03
4.329004e−03
4.329004e−03
4.329004e−03
2.778430e−03
2.164502e−03
p−value(wilcox test)
